# Effects of Thymoquinone on Urotensin-II and TGF-β1 Levels in Model of Osteonecrosis in Rats

**DOI:** 10.3390/medicina59101781

**Published:** 2023-10-06

**Authors:** Mehmet Yilmaz, Recep Dokuyucu

**Affiliations:** 1 Department of Orthopedic Surgery, 25 Aralik State Hospital, Gaziantep 27090, Turkey; doctor_yilmaz@hotmail.com; 2 Department of Physiology, Private Fizyoclinic Wellness Center, Gaziantep 27560, Turkey

**Keywords:** thymoquinone, nutrition, osteonecrosis, rat, Urotensin-II, transforming growth factor-beta-1

## Abstract

*Objectives*: We aimed to investigate the therapeutic effects of thymoquinone (TMQ) treatment in osteonecrotic rats by evaluating protein levels, osteonecrosis (ON) levels, fatty acid degeneration, oxidative status, and plasma levels of Urotensin-II (U-II) and transforming growth factor-beta (TGF-β1). *Materials and Methods*: 40 weight-matched adult male Wistar rats were grouped as control (*n* = 10), methylprednisolone acetate (MPA) (*n* = 10), thymoquinone (TMQ) (*n* = 10), and MPA + TMQ (*n* = 10). To induce ON, 15-week-old animals were subcutaneously injected with MPA at a dose of 15 mg/kg twice weekly for 2 weeks. TMQ was injected into 15-week-old rats via gastric gavage at a dose of 80 mg/kg per day for 4 weeks. The rats in the MPA + TMQ group were administered TMQ 2 weeks before the MPA injection. At the end of the treatments, cardiac blood samples and femur samples were collected for biochemical and histological evaluations. *Results*: In the control and TMQ groups, no ON pattern was observed. However, in tissues exposed to MPA, TMQ treatment resulted in significantly decreased ON levels compared to the MPA group. The number of cells that were positive for 8-OHdG and 4-HNE was significantly lower in the MPA + TMQ group than in the MPA group (*p* < 0.05). In terms of TGF-β1 and U-II levels, we observed that both TGF-β1 (367.40 ± 23.01 pg/mL vs. 248.9 ± 20.12 pg/mL) and U-II protein levels (259.5 ± 6.0 ng/mL vs. 168.20 ± 7.90 ng/mL) increased significantly in the MPA group compared to the control group (*p* < 0.001). Furthermore, TGF-β1 (293.50 ± 14.18 pg/mL) and U-II (174.80 ± 4.2 ng/mL) protein levels were significantly decreased in the MPA + TMQ group compared to the MPA group (*p* < 0.05 and *p* < 0.01, respectively). There was a statistically positive correlation (*p* < 0.05) between the TGF-β1 and U-II protein levels in all groups (*p* = 0.002, r_control_ = 0.890; *p* = 0.02, r_TMQ_ = 0.861; *p* = 0.024, r_MPA+TMQ_ = 0.868) except for the MPA group (*p* < 0.03, r_Medrol_ = −0.870). *Conclusions*: As far as we know, this is the first study to demonstrate the curative functions of TMQ on ON by causing a correlated decrease in the expression of U-II and TGF-β1 in the femoral heads of rats.

## 1. Introduction

Osteonecrosis, also known as avascular or aseptic necrosis, is defined as bone cell death that occurs following reduced bone blood circulation due to a traumatic or nontraumatic cause. The most common sites of osteonecrosis are the femoral head, shoulder, knee, and ankle regions. This disease, which mostly occurs in active and productive individuals, not only negatively affects the life of the person, but also imposes a heavy burden on society. The commonest reason for osteonecrosis is trauma that causes damage to vascular structures and impaired blood flow. Although there are many nontraumatic causes, 90% of these causes are corticosteroid use [1,2,3,4]. Due to the COVID-19 epidemic, osteonecrosis started to be seen very frequently [5,6,7]. Many hypotheses have been put forward for the pathogenesis. These include direct cellular toxicity, oxidative stress, coagulopathic events, hyperlipidemia, fat embolism, vascular anomalies and disorders, increased bone marrow pressure [5,6,7].

The main goal in osteonecrosis treatment is the preservation of the femoral head. However, most patients apply to the physician in the advanced stages of the disease. Although early diagnosis is possible in the management of this process, the treatment is not yet satisfactory [8,9,10]. The morbidity is high due to the progressive course of the disease. For this reason, research on understanding the etiology and treatment has been gaining momentum in recent years. Medical and surgical treatments have been shown to provide symptomatic relief, but early intervention is crucial for successful results in joint-preserving procedures [8,9,10]. Early diagnosis and preservation of the hip joint, which are the main treatment principles in osteonecrosis, suggest surgical treatment options in patients at risk of progression. Nonoperative approaches are weight-bearing reduction, pharmacological agents, electromagnetic and ultrasonography stimulation, ESWT (extracorporeal shock wave therapy), and hyperbaric oxygen therapy [1,3].

Thymoquinone (TMQ) is the main bioactive compound of Nigella Sativa seeds, a medicinal plant used in the treatment of many diseases [11]. The taking of herbal medicines in the treatment of various diseases is increasing day by day. Plants contain chemicals that provide many benefits, and research in the field of health and the allocated resources encourage the use of herbal products in both the treatment of diseases and preventive medicine [12]. The protective effects of thymoquinone have been demonstrated in many disease models, including cancer, autoimmune disease, muscle and joint diseases, neurological diseases, endocrine diseases, and asthma [13,14,15]. The vast majority of studies have focused on these two effects of thymoquinone. In a testicular ischemia-reperfusion study in rats, it was reported that thymoquinone acts by reducing apoptosis, oxidative stress, and lipid peroxidation [16]. Various research studies have reported the positive effects of thymoquinone on bone and cartilage tissues. TMQ exerts an osteoprotective effect by inhibiting osteoclasts and reducing bone matrix resorption [13,14,17,18]. In an animal study, TQ was shown to increase bone healing in important vital and reproductive organs without significant side effects [19]. It has been shown that TQ can be used as a safe and effective treatment because of its capacity for anti-inflammatory, antioxidant, and anticatabolic effects in the treatment of joint diseases (osteoarthritis and rheumatoid arthritis) [17,20].

Urotensin-II (U-II) is a polypeptide molecule with neurohormone-like activity and is known to be expressed mainly in the cardiovascular, pulmonary, renal, and central nervous systems. ^14^ Although UT-II was generally identified as the most potent vasoconstrictor in previous studies, later studies have shown that UTS2 has many biological activities in processes such as proliferation, fibrosis, and inflammation. U-II was reported to induce collagen synthesis in cardiac fibroblasts via TGF-β1 in a rat model [21]. In a study on rats, it was reported that treatment with U-II raised the synthesis of type-I collagen in vascular smooth muscle cells using the transforming growth factor-beta (TGF-β1) signaling pathway [22]. Thus, it can be thought that TGF-β1 and U-II have important effects on the formation of vascular fibrosis. In addition, in a study conducted in osteoarthritis patients, it was reported that TGF-β1 and U-II act together in the formation of synovial fibrosis due to the high levels of U-II in the knee joint fluid of the patients [23]. On the other hand, in the literature about osteoarthritis, TGF-β1 was shown to have an important protective role against cartilage loss in animal models [24,25].

Osteonecrosis has started to be seen very frequently due to the steroids widely used in the treatment of COVID-19. In our study, we aimed to investigate the therapeutic effects of TMQ treatment in osteonecrotic rats by evaluating protein levels, ON levels, fatty acid degeneration, oxidative status, and plasma levels of Urotensin-II (U-II) and transforming growth factor-beta (TGF-β1).

## 2. Materials and Methods

### 2.1. Ethics Statement and Experimental Design

All experiments were approved by the Mustafa Kemal University Local Ethics Committee, Hatay, Turkey (Approval no: 2014/7-1, date: 22 July 2014). In this study, male Wistar albino rats (250–300 g) were obtained from Mustafa Kemal University from the Faculty of Medicine Experimental Animal Center. The rats to be used in the experiment were kept in room temperature conditions in a dark-and-light environment for 12 h. During the experiment, these rats were fed with normal mouse chow and water. Throughout the experiment, rats were provided with conditions according to the international laboratory animal ethical guidelines.

A total of 40 weight-matched adult male Wistar rats were grouped as control (*n* = 10), methylprednisolone acetate (MPA) (*n* = 10), thymoquinone (TMQ) (*n* = 10), and MPA + TMQ (*n* = 10). To induce ON, 15-week-old animals were subcutaneously injected with MPA (Sigma-Aldrich, St. Louis, MO, USA) at a dose of 15 mg/kg twice weekly for 2 weeks [26]. TMQ (Sigma-Aldrich) was injected into 15-week-old rats via gastric gavage at a dose of 80 mg/kg per day for 4 weeks [8,27].

The rats in the MPA + TMQ group received TMQ two weeks prior to the MPA injection. After the treatment period, all the animals were put to sleep with anesthesia (12 mg/kg xylazine and 80 mg/kg ketamine i.p.). Subsequently, samples of cardiac blood and femur were collected for biochemical analysis and histological examination.

### 2.2. Biochemical Analyses

TGF-β1 and Urotensin-II plasma levels were calculated with ELISA kit. Measurable levels of TGF-β1 and U-II ranged from 5 to 1000 ng/mL.

### 2.3. Histopathological and Immunohistochemical Analysis

Bilateral femoral specimens were placed on paraffin blocks after fixation (10% formalin) for one day and decalcification (EDTA). The samples were then prepared for immunohistochemical (IHC) and histological analysis, which involved Hematoxylin-eosin staining. The assessment of the histopathological changes was carried out as per the criteria previously established by Nozaki and his colleagues [28]. The main criteria of ON are osteocyte necrosis and fatty degeneration, which were analyzed according to Nozaki et al. [28]. By utilizing 8-hydroxy-2′-deoxyguanosine (8-OHdG) and 4-hydroxy-2-nonenal (4-HNE) and IHC assays, DNA damage and lipid peroxidation, respectively, were measured in the bone tissue. To detect oxidative stress, an immunohistochemical analysis was conducted using the avidin-biotin immunoperoxidase technique with 8-hydroxy-2′-deoxyguanosine (anti-8-OHdG) and 4-hydroxy-2-nonenal (anti-4–HNE) polyclonal antibodies. The anti-8-OHdG antibody was used because when oxidative stress increases due to active oxygen species, 8-OHdG forms in DNA and is detected in the nucleus. For 8-OHdG, observations were scored on a three-point scale: (1) observation areas were unstained; (2) some myelocytes were stained; and (3) all bone marrow cells were positively stained. The anti-4-HNE antibody detects 4-HNE, a secondary product of oxidation of w-6 polyunsaturated fatty acids found particularly around adipose cells. For 4-HNE, findings were scored using a three-point scale: (1) observation areas were unstained; (2) only adipose cell borders were stained; and (3) bone marrow cells were also positively stained. The intensity of staining was considered when assessing the staining for 4HNE and 8-OHdG. To evaluate the findings objectively, the staining of cells and peripheral structures such as blood vessels, adipocytes, bone marrow cells, and trabeculae was assessed using a three-point staining scale (negative, positive, and intensely positive). The average total score of each group was calculated using a 3-point method [28].

### 2.4. Statistical Analysis

GraphPad Prism 9.0 (GraphPad Software, San Diego, CA, USA) program was used for statistical analysis. Student’s *t* and Pearson correlation tests were used to compare the weights and blood sugar levels of the rats in the group. A *p*-value below 0.05 was considered statistically significant.

## 3. Results

### 3.1. Histopathological Examination

**Figure 1:**
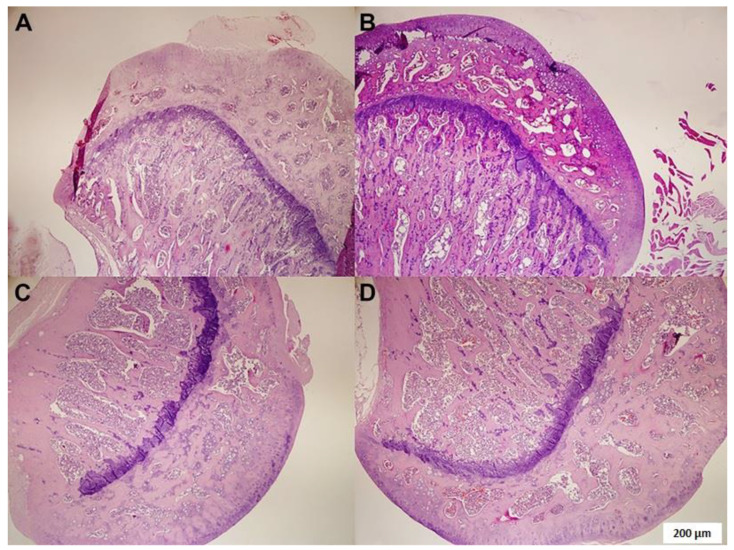
Staining of femur specimens with hematoxylin-eosin. Control (**A**); MPA (**B**); TMQ (**C**); and MPA + TMQ (**D**). (Methylprednisolone acetate (MPA); thymoquinone (TMQ), ×100; scale bar: 200 µm.)

Fatty degeneration and ON grades were calculated using histopathological evaluation and hematoxylin-eosin staining (Figure 1, Table 1). There was a significant reduction in fat degeneration in the MPA + TMQ group compared to the control and MPA-treated groups (*p* < 0.05, Table 1). MPA-treated tissue had multiple unstained holes and more intense dark-blue hematoxylin staining due to high lipid steatosis due to increased oxidation within the tissue compared to other groups in terms of increased fat degeneration.

There was no ON pattern in the control and TMQ groups. In tissues exposed to MPA, the application of TMQ treatment resulted in a significant reduction in ON levels compared to the MPA-alone group, as evidenced by *p* < 0.05, Table 1, and Figure 1. This significant alteration was also observed in the H&E-stained tissues treated with MPA, which showed a loss of tissue integrity and cellular mass due to an increase in the ON pattern (as shown in Figure 1B,D). There was no significant difference between the TMQ group and the control group. No new bone formation was observed in the rats. As per the classification referenced in the literature, while the incidence of osteonecrosis was 50% in the MPA group, this rate was observed to be lower in the MPA + TMQ group.

### 3.2. Oxidative Evaluations

**Figure 2:**
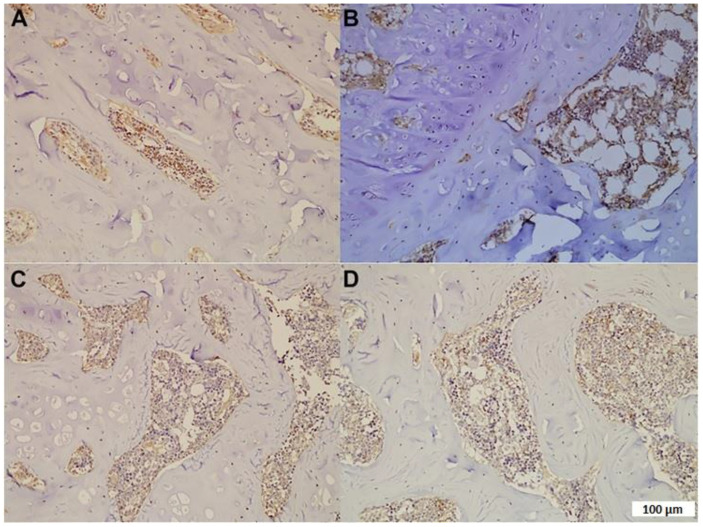
Immunohistochemical staining of femur specimens (4-hydroxy-2-nonenal (4-HNE); anti-8-hydroxy-2′-deoxyguanosine (8-OHdG).) Control (**A**); MPA (**B**); TMQ (**C**); and MPA + TMQ (**D**). (Methylprednisolone acetate (MPA); thymoquinone (TMQ), ×100; scale bar: 100µm.)

According to the categorization mentioned in the studies, the MPA group had an osteonecrosis occurrence of 50%, whereas this percentage was found to be lower in the MPA + TMQ group (*p* < 0.05). In addition to these findings, in Figure 2B, due to increased lipid steatosis, it is clearly seen that large holes were formed in MPA-treated tissue with increasing 8-OHdG- and 4-HNE-positive cell numbers. Figure 2D illustrates a significant decrease in the count of 8-OHdG- and 4-HNE-positive cells in the MPA+ TMQ group in comparison to the MPA group (*p* < 0.05, see Table 1). The TMQ and control groups were similar.

### 3.3. Expression of U-II and TGF-β1 

**Figure 3:**
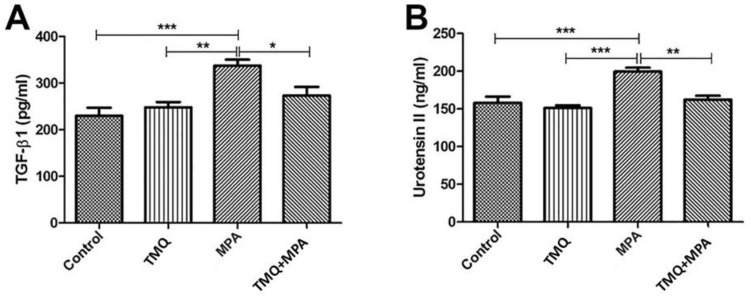
Comparison of TGF-β1 (**A**) and U-II (**B**) protein levels measured by ELISA. Methylprednisolone acetate (MPA); thymoquinone (TMQ). ***: *p* < 0.001; **: *p* < 0.01; *: *p* < 0.05.

The levels of TGF-β1 and U-II calculated using the ELISA test are shown in Figure 3. In terms of TGF-β1 and U-II levels, we observed that both TGF-β1 (367.40 ± 23.01 pg/mL vs. 248.9 ± 20.12 pg/mL) and U-II protein levels (259.5 ± 6.0 ng/mL vs. 168.20 ± 7.90 ng/mL) increased significantly in the MPA group compared to the control group (*p* < 0.001). In addition, TGF-β1 and U-II levels were also significantly higher in the MPA group compared to the TMQ treatment group (268.20 ± 9.75 pg/mL, *p* < 0.01, and 140.80 ± 6.24 ng/mL, *p* < 0.001, respectively). Furthermore, TGF-β1 (293.50 ± 14.18 pg/mL) and U-II (174.80 ± 4.2 ng/mL) protein levels were significantly decreased in the MPA + TMQ group compared to the MPA group (*p* < 0.05 and *p* < 0.01, respectively). 

**Figure 4:**
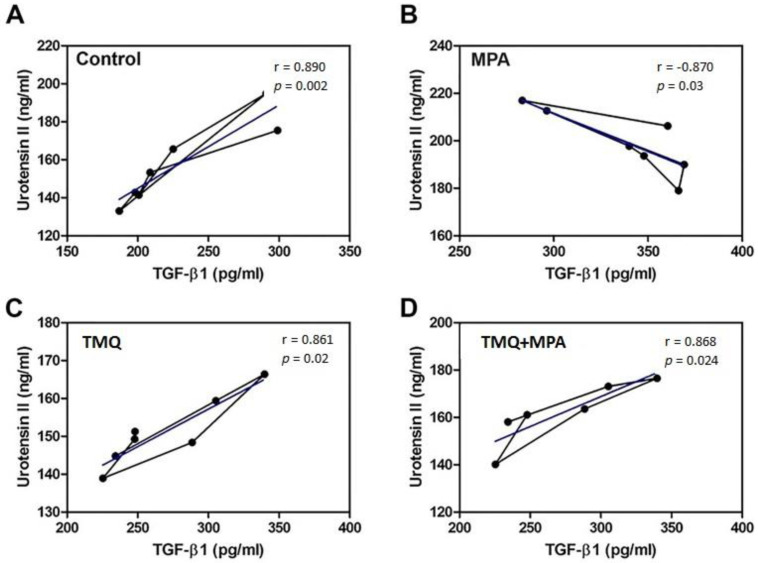
The correlation analysis between the protein levels of TGF−β1 and U−II in control (r = 0.890, *p* = 0.002) (**A**), MPA (r = −0.870, *p* = 0.03) (**B**), TMQ (r = 0.861, *p* = 0.02) (**C**), and MPA + TMQ (r = 0.868, *p* = 0.024) (**D**) groups. Methylprednisolone acetate (MPA); thymoquinone (TMQ).

A positive statistical correlation (*p* < 0.05) was observed between the TGF-β1 and U-II protein levels across all groups (see Figure 4A,C,D), with r_control_ = 0.890, r_TMQ_ = 0.861, and r_MPA+TMQ_ = 0.868. A negative statistical correlation was observed between the TGF-β1 and U-II protein levels in the MPA group (refer to Figure 4B, r_Medrol_ = −0.870) (*p* < 0.05).

## 4. Discussion

Our study is the first in the literature to evaluate the effect of thymoquinone (TMQ) on the osteonecrosis model and the role of TGF-B and U-II in osteonecrosis. In our study, we performed histopathological, immunohistochemical, and biochemical analyses in order to evaluate the efficacy and systemic effects of TMQ treatment in osteonecrotic areas by utilizing the antioxidant, antiosteoclastic, osteoblastic, and hypolipidemic effects of TMQ treatment on a steroid-induced osteonecrosis model in rats.

It has been known for a long time that the most important cause of ON occurring without trauma is the use of glucocorticoids (GCs) for therapeutic purposes against the chronic inflammation seen in diseases that cause circulatory disorders. However, the most frequently affected area in ON caused by GCs is the femoral head [8,9,29]. Although studies in the literature indicate that there is no direct relationship between the pathophysiology of ON and GC treatments given for another disease, there is an experimental model stating that there is a strong relationship between GC implementation and the development of ON. In a rabbit model of ON, a single implementation of high-dose GC was reported to cause ON in different animal bones [30]. Moreover, in a different ON model, implementation of high-dose GC was reported to cause a decrease in bone blood flow, leading to the onset of ON [31]. It was noted that in the rabbit model of MPA-induced ON, LDL cholesterol levels of circulatory-related parameters were significantly higher, leading to decreased bone blood circulation [32].

In a study, it was stated that treatment with MPA led to circulatory hypocoagulability in healthy individuals [33]. However, hypercoagulability leading to thrombus development was demonstrated in a rabbit model with GC-induced osteonecrosis [34]. These conflicting results in the literature may be due to the different effects of GCs based on samples from healthy and osteonecrotic specimens and their concomitant pathophysiologies [35]. In our study, we showed that osteonecrosis occurred with MPA being given to healthy rats.

Studies in the literature have shown that treatment with anticoagulant and/or lipid-lowering agents prevents the progression of ON in animal models of GC-induced ON [8,9,10,36,37,38]. In the ON rabbit model by Ichiseki et al. [39], it was reported that GC treatment caused oxidative damage to DNA in the initial stage of ON development. In support of this study, several studies have reported that GC treatment downregulates Cbfa1, which increases the remodeling factor and bone formation expression, thus causing the dysfunction of osteoblast and oxidative stress-induced apoptosis [2,40]. In addition, it was reported that antioxidant treatments improved bone metabolism and optimized ON-induced oxidative stress in MPA-induced ON models [40,41]. Weinstein stated that the most important cause of osteonecrosis is high osteoblast and osteocyte apoptosis levels, which lead to impaired bone circulation due to reduced angiogenic factors [2]. Based on studies conducted with thymoquinone in the literature, we have shown the positive effects of thymoquinone in the treatment of ON in our study by drawing from the antioxidant, antiosteoclastic, osteoblastic, and hypolipidemic effects of thymoquinone.

In our study, we demonstrated the effects of TMQ, an anti-inflammatory, antioxidant, antiosteoclastic, osteoblastic, and hypolipidemic agent, on GC-induced ON in rats. Significant reductions in both fat degeneration and ON levels were observed in the histopathological evaluations. In addition, the treatment with TMQ also diminished the levels of oxidative stress by lowering the counts of both 8-OHdG- and 4-HNE-positive cells in the femoral head, which are indicative of cellular DNA damage and lipid peroxidation levels, respectively. In a literature study of GC-induced osteonecrosis in rabbits, injection of ALA was reported to significantly reduce the ON pathogen through the improvement of vascular blood flow and the inhibition of oxidative stress [42]. In addition, in our previous study, we suggested that alpha lipoic acid agent with antioxidant and anti-inflammatory properties can be used in the treatment of ON [8].

Thanks to its anti-inflammatory, antioxidant, antiosteoclastic, osteoblastic, and hypolipidemic mechanism of action, TMQ is recommended in the treatment of many diseases, such as diabetes mellitus, cancer, autoimmune diseases, heart diseases, hypertension, and neurodegenerative disorders, as a therapeutic option [43,44,45]. To elucidate the mechanisms underlying the multiple protective effects of TMQ, we evaluated the levels of TGF-β1 and U-II using ELISA. Studies in the literature have shown that TGF-β1 and UT-II have many biological activities in processes such as proliferation, fibrosis, and inflammation [9,46]. As shown in Figure 1 of our study, TMQ treatment given after MPA-induced ON development significantly reduced fatty degeneration, osteonecrosis, 4-HNE, and 8-OHdG levels. In addition, as shown in Figure 2, we found that TGF-β1 and UT-II levels were strongly correlated with each other in the control and TMQ-only groups. On the other hand, the strong correlation between the TGF-β1 and UT-II levels appeared to be impaired in the MPA-only group. However, as a result of TMQ treatment, this strong positive relationship between TGF-β1 and UT-II levels was re-established in the MPA + TMQ group. Studies in the literature indicate that U-II upregulation is closely related to the TGF-β1 pathway and support our results [22]. In addition, as demonstrated in our study, increased levels of both TGF-β1 and UT-II proteins in the synovial fluids of patients with OA and osteoradionecrosis would be beneficial in terms of knowing their roles in the pathophysiology of such diseases [23,47]. Research indicates that the TGF-β1 signaling pathway is essential for joint formation. However, its elevated levels in adulthood could potentially contribute to degenerative conditions such as osteoarthritis and osteonecrosis through synovial fibrosis [48,49]. In addition, promising new therapeutic interventions may emerge for the treatment of osteo-degenerative disorders by modifying the expression of proteins, such as U-II, that are involved in the TGF-β1 signaling pathway.

Our research does have certain limitations, primarily including the scarcity of studies employing rats in the osteonecrosis model. Various animal species, including rabbits, dogs, birds, and sheep, have been used as experimental models of steroid-induced ON. Studies using rats have shown that the pathology of osteonecrosis in this model more closely resembles the pathology of osteonecrosis in humans than in rabbits [4]. This is also one of the strengths of our study. Other limitations are that we used fewer animals and only analyzed one blood draw. In order to comply with the 3R rule accepted in scientific studies on animals, less stimulation was performed for fewer animals in suitable conditions. Intraperitoneal and intramuscular injections in the first week of our study and oral gavage implementation throughout the study caused enough stress for the rats. For this reason, biochemical analysis could be performed at the end of the study. Another limitation of our study was the administration of a single daily dosage of TMQ in our study. The effects of dosing need to be further investigated to determine the optimal dose for reducing the incidence of steroid-induced ON. In addition, the 4-week observation period designed in our study could be shortened to determine the short-term efficacy of TMQ therapy. 

## 5. Conclusions

In conclusion, we tried to demonstrate the therapeutic effects of TMQ, which has antioxidant, antiosteoclastic, osteoblastic, and hypolipidemic effects with correlated decreases in U-II and TGF-β1 expression, in a model of osteonecrotic rats. As far as we know, this is the first study to demonstrate the curative functions of TMQ in ON by causing a correlated decrease in the expression of U-II and TGF-β1 in the femoral heads of rats. Further studies are needed to elucidate the link between TMQ therapy and the TGF-β1/U-II signaling pathways at the molecular level and to find the curative potential of this approach for ON patients.

## Figures and Tables

**Table 1:** The comparison of histopathology and immunohistochemistry results between the groups.

	Control (*n* = 10)	MPA (*n* = 10)	TMQ (*n* = 10)	MPA + TMQ (*n* = 10)
Fatty degeneration	0.01 ± 0.01	1.53 ± 0.1	0.30 ± 0.1	0.76 ± 0.2 ^b^*
Osteonecrosis	0.00 ± 0.01	3.50 ± 0.29	0.00 ± 0.01	2.13 ± 0.35 ^b^*
4-HNE	0.97 ± 0.65	3.69 ± 0.22 ^a^*	1.29 ± 0.30	1.75 ± 0.28 ^b^*
8-OHdG	0.95 ± 0.46	3.50 ± 0.34 ^a^*	1.37 ± 0.19	1.55 ± 0.41 ^b^*

*: *p* < 0.05. Methylprednisolone acetate (MPA); thymoquinone (TMQ); 4-hydroxy-2-nonenal (4-HNE); anti-8-hydroxy-2′-deoxyguanosine (8-OHdG). ^a^ Control vs. MPA; ^b^ MPA vs. MPA + TMQ.

## Data Availability

The data that support the findings of this study are available from the corresponding author upon reasonable request.
